# Engineering Bacterial Extracellular Vesicles as Nanoweapons to Fight against Bacterial Infections

**DOI:** 10.34133/research.1135

**Published:** 2026-02-05

**Authors:** Yejiao Shi, Yuting Li, Zhinan Liu, Xiangxiang Kong, Xiaochun Hu, Xi Liu, Cuiping Zhang, Honggang Hu

**Affiliations:** ^1^ Institute of Translational Medicine, Shanghai University, Shanghai 200444, China.; ^2^ Institute of Translational Medicine, Shanghai Jiao Tong University, Shanghai 200030, China.; ^3^ Shanghai Integration and Innovation Center of Marine Medical Engineering, Shanghai 200444, China.; ^4^ School of Medicine, Shanghai University, Shanghai 200444, China.; ^5^ Medical Innovation Research Department, PLA General Hospital and PLA Medical College, Beijing 100143, China.; ^6^ School of Pharmacy, Chengdu Medical College, Chengdu 610083, China.

## Abstract

The overuse and misuse of antibiotics have led to widespread resistance in bacteria, which makes infections difficult to treat. The insufficient prevention measures, limited treatment options, and delayed antibiotic developments call for immediate global actions to discover effective and safe treatments for bacterial infections. Over the past decades, more and more studies have found that bacterial extracellular vesicles (BEVs) secreted by bacteria with nanoscale size, lipid bilayer structure, pathogen-associated molecular patterns, and inherent bioactive substances are the ideal candidates for bacterial infection treatment. Meanwhile, advanced engineering approaches have further endowed these BEVs with more customizable properties to effectively fight against bacterial infections. Herein, the present review begins with an overview of the biogenesis and biocomponents of BEVs to better comprehend their bioactivities against bacterial infections. Their isolation and engineering approaches are then introduced, with an emphasis on the diverse genetic, physical, and chemical strategies to functionalize them with desirable capacities for the optimal treatment of bacterial infections. Recent advances in exploring the natural BEVs as antibacterial and antiadhesion agents, as well as the engineered BEVs as vaccine antigens, vaccine adjuvants, and delivery nanocarriers, are expounded successively. Discussions on the new trend of engineering BEVs as nanoweapons to combat bacterial infections, in terms of advantages and challenges, are provided at the end to expedite these BEV-based therapeutic modalities for bacterial infections from bench to bedside.

## Introduction

Bacterial infections are diseases that can occur in almost every part of the human body, such as the skin, lung, brain, and even blood [1–4]. It causes approximately 7.7 million deaths each year and poses persistent threats to public health globally [5]. Currently, only a limited number of vaccines are available for the prevention of bacterial infections. Antibiotics remain the most common therapeutics used in clinical practice, being applied not only for the treatment of conventional bacterial infections, but also for the prevention of postoperative bacterial infections [6]. Unfortunately, most antibiotics still suffer from the rapid clearance rate, limited penetrating efficiency, and insufficient targeting capacity in vivo. To maintain their effective concentrations at the infection site, antibiotics are administered at high intensities and dosages, which progressively drive the emergence of bacterial resistance. The enhanced resistance requires intense administration, accelerating the spread of resistance in turn [7]. Although novel antibiotics have been discovered, the speed at which they are brought to market and applied to patients is too slow to meet the challenges posed by the resistant bacterial infections [8–12]. The lack of prevention measures, limited treatment options, as well as delayed antibiotic developments have led to the urgent demand of effective and safe treatments for bacterial infections.

Inspired by nature, the metabolic products derived from bacteria have been extensively explored for the effective and safe treatment of bacterial infections [13]. Apart from the most famous antibacterial peptides, the bacterial extracellular vesicles (BEVs) that are secreted by bacteria have also attracted tons of interest [14–17]. These BEVs with lipid bilayer nanostructures are capable of transferring the diverse bioactive molecules from bacteria to the host [18]. As such, they have been considered important messengers for interkingdom communication, being responsible for the progression of bacterial infections and even resistance [19–21]. Nonetheless, empowered by the advanced technologies, these BEVs have also been deeply dug as nanoweapons to fight against bacterial infections.

Owing to their structural and componential similarities to bacteria, the natural BEVs have firstly been exploited for the bacterial infection treatment directly [22]. Since they inherit various endogenous antibacterial cargoes, including the hydrolases, peptides, and metabolites from their parental bacteria, the BEVs have been directly used as antibacterial agents to effectively kill the pathogenic bacteria [23,24]. Besides, their inherited surface proteins, such as the adhesins, have also enabled their direct usage as antiadhesion agents to competitively inhibit the adhesion and infection of the pathogenic bacteria to the host [25].

Benefiting from the emerging functionalization approaches, the engineered BEVs have also been constructed for bacterial infection treatment further [26]. Based on the genetic or chemical engineering approaches, the surface components of BEVs, such as the inherited pathogen-associated molecular patterns (PAMPs) and pathogen-specific antigens, have been rationally modified. Therefore, the engineered BEVs have been exploited as either vaccine antigens or adjuvants with the appropriate immunostimulatory effectiveness for the effective and safe treatment of bacterial infections [27]. Besides, by the genetic or physical engineering approaches, the exogenous cargoes of BEVs have also been rationally loaded [28–33]. Therefore, the engineered BEVs have been exploited as nanocarriers as well with the desired targeting and penetrating capacities for the optimal treatment of bacterial infections.

**Fig. 1.**
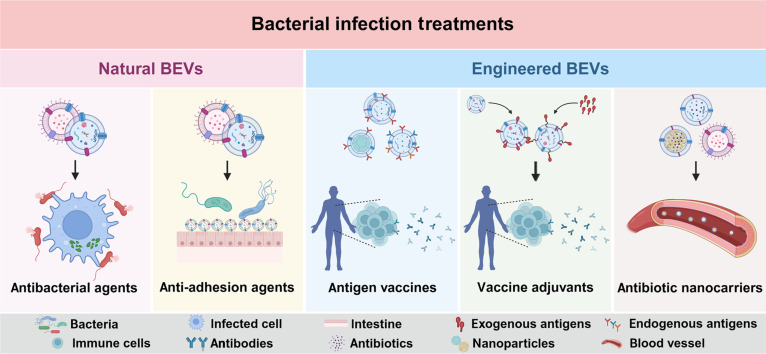
The 5 therapeutic roles acted by bacterial extracellular vesicles (BEVs) for the treatment of bacterial infections. The natural BEVs can act as antibacterial agents to inhibit the growth and reproduction of bacteria, or act as antiadhesion agents to reduce the adhesion of bacteria for the bacterial infection treatment. The engineered BEVs can serve directly as antigen vaccines, or act as vaccine adjuvants to enhance the protective immune efficacy of vaccines, or act as antibiotic carriers to increase the utilization rate of antibiotics for bacterial infection treatment. Created in BioRender.

With more and more BEVs being reported for bacterial infection treatment, a systematic search was performed to identify the relevant studies published up to 2025. The primary databases used were PubMed, Web of Science, and Scopus, and the keywords used were “bacterial extracellular vesicles or bacterial outer membrane vesicles” as well as “bacterial infections or antibacterial”. Approximately 2,900, 2,000, and 1,600 corresponding articles in English were retrieved in the 3 databases, respectively. With respect to the different functional roles of BEVs as antibacterial agents, antiadhesion agents, antigen vaccines, vaccine adjuvants, and antibiotic nanocarriers for the bacterial infection treatment, the landmark studies with historical progresses were selected as the classic cases. Although several reviews have preliminarily summarized the antibacterial applications of BEVs as either vaccines and nanocarriers, nanocarriers, or vaccines, respectively [14,24,34–36], a comprehensive review systematically elaborating on all their 5 different functional roles and relevant engineering approaches is still lacking. Herein, the biogenesis and biocomponents of BEVs were firstly overviewed to facilitate the understanding of their bioactivities against bacterial infections. The isolation techniques and engineering strategies were then compared to underline the advantages and limitations of each approach in functionalizing the BEVs for the optimal bacterial infection treatment. Based on these fundamental cognitions, recent advances in exploiting the BEVs as antibacterial agents, antiadhesion agents, vaccine antigens, vaccine adjuvants, and delivery nanocarriers were expounded one by one (Fig. 1). The unique superiorities and future challenges in engineering the BEVs as nanoweapons to conquer the bacterial infections were discussed by the end. The insights of the presented review would guide the future developments and clinical applications of the BEV-based therapeutic modalities for the effective and safe treatment of bacterial infections.

## Biogenesis and Biocomponent of BEVs

**Fig. 2.**
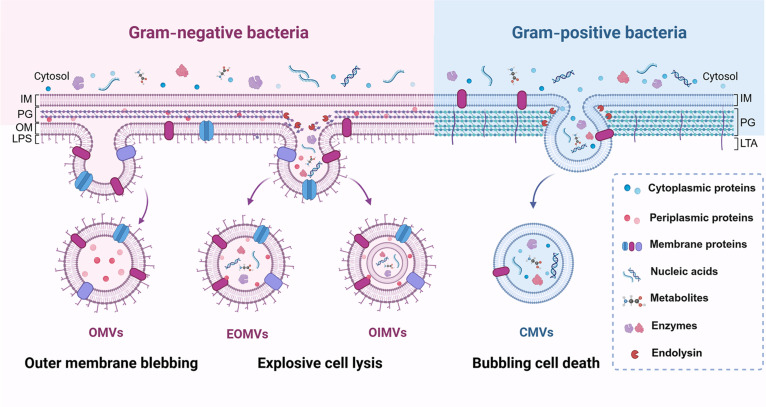
The biogenesis and biocomponents of BEVs. Gram-negative bacteria generate outer membrane vesicles (OMVs) via outer membrane blebbing, while generating explosive outer membrane vesicles (EOMVs) as well as outer inner membrane vesicles (OIMVs) via the explosive cell lysis. By contrast, gram-positive bacteria generate cytoplasmic membrane vesicles (CMVs) via the bubbling cell death. These 4 different BEVs are collectively referred to as the BEVs in the present review. The different biogenetic mechanisms of BEVs lead to their distinct compositions, including lipopolysaccharide (LPS), proteins, nucleic acids, and metabolites. Created in BioRender.

Based on the staining properties, bacteria are classified as gram-negative bacteria and gram-positive bacteria. The gram-negative bacteria have an inner membrane (IM), a thin peptidoglycan (PG) layer, and an outer lipid membrane displaying lipopolysaccharide (LPS) (Fig. 2), while gram-positive bacteria have only the IM and a thick PG layer displaying lipoteichoic acid (LTA) (Fig. 2). Despite these structural differences, both gram-negative and gram-positive bacteria can secrete BEVs spontaneously without any energy consumption [37]. The secretion processes and mechanisms of BEVs are highly dependent on the surrounding environment of bacteria. In terms of the different modes the BEVs generate, their components vary quite a lot. Since the structure and composition of BEVs are the basis of their biofunctions, their biogenesis and biocomponents were firstly introduced below to better comprehend their applications for bacterial infection treatment.

### BEVs derived from gram-negative bacteria

BEVs were firstly identified from gram-negative bacteria in the 1960s, when the EVs released in medium were found during the normal growth of *Pseudomonas aeruginosa* (*P. aeruginosa*) [38]. Since then, other gram-negative bacteria, such as *Salmonella* sp. [39], *Shigella* sp. [40], *Helicobacter pylori* (*H. pylori*) [41], and *Acinetobacter baumannii* (*A. baumannii*), have also been gradually found to secrete EVs [42]. These EVs with a diameter ranging from 10 to 300 nm were mainly released by gram-negative bacteria via two pathways (Fig. 2) [43]. One pathway is the outer membrane blebbing induced by the insertion of hydrophobic molecules or the unstable biosynthesis of PG, generating the classical outer membrane vesicles (OMVs) [44]. The other pathway is the explosive cell lysis mediated by the weakening of the PG layer via endolysins and the protrusion of IM to the pericyte, generating the explosive outer membrane vesicles (EOMVs) as well as the outer inner membrane vesicles (OIMVs), respectively [44]. These two pathways have been further reported to coexist during the BEV generation period, rather than being independent from each other [45].

The different biogenetic mechanisms of BEVs lead to their distinct compositions (Fig. 2). Typically, the OMVs only contain the outer membrane proteins and lipids due to the unbroken IM during the generation process [46]. By contrast, the EOMVs and OIMVs not only contain these outer membrane components, but also contain the cytoplasmic membrane proteins, nucleic acids, metabolites, and even virulence factors randomly because of the damaged PG layer during the generation process [43].

### BEVs derived from gram-positive bacteria

BEVs derived from gram-positive bacteria were not reported until 1990, nearly 30 years after the discovery of gram-negative bacteria-derived BEVs [39]. The comparatively fewer research interests in gram-positive bacteria-derived BEVs are largely due to their thick cell walls, which have been considered as physical barriers limiting the release of BEVs [47]. With the vesicle-like blisters being observed on the surface of *Bacillus*, growing evidence has been reported that BEVs were capable of passing through the thick cell walls of gram-positive bacteria to reach the extracellular space. For instance, BEVs with a diameter ranging from 20 to 100 nm were demonstrated to be released from gram-positive *Staphylococcus aureus* (*S. aureus*) in 2009 [48]. Although several hypotheses on the release mechanism have been proposed, it is now commonly believed that the degradation of the PG layer induced bubbling cell death is the main cause of the generation of the cytoplasmic membrane vesicles (CMVs) (Fig. 2) [49].

As such, CMVs contain abundant cytoplasmic substances such as plasma membrane proteins, nucleic acids, and endolysins [50]. Unlike gram-negative bacteria-derived BEVs, BEVs derived from gram-positive bacteria do not contain LPS but possess LTA, which is also an important PAMP that can cause the innate immune response [26,51,52]. Apart from LPS, the differences in other PAMPs such as the lipoprotein, PG, DNA, and RNA that are included in gram-negative and gram-positive BEVs ultimately lead to the diverse biofunctions of BEVs for bacterial infection treatment.

## Isolation and Engineering of BEVs

### Isolation techniques of BEVs

To effectively collect BEVs with structural and functional integrity, diverse isolation techniques have been developed based on the specific properties of BEVs. Among them, the commonly utilized techniques include ultracentrifugation (UC), density gradient centrifugation (DGC), ultrafiltration (UF), size exclusion chromatography (SEC), protein precipitation, and affinity separation [53]. As summarized in Table 1, these techniques have their own advantages and limitations.

**Table 1.** The conventionally used techniques to isolate BEVs: their mechanisms, advantages, as well as limitations

Techniques	Mechanisms	Yield of Pure BEVs	Advantages	Limitations	References
Ultracentrifugation	Based on the difference in the density of BEVs	~10^7^–10^9^ particles /μg protein	Simple process; good repeatability	Low purity	[180]
Density gradient centrifugation	Based on the difference in the density of BEVs	~10^9^–10^10^ particles /μg protein	High purity	High costs; time-consuming	[55]
Ultrafiltration	Based on the difference in the size of BEVs	~10^8^–10^9^ particles /μg protein	Simple process	Low purity; low efficiency.	[56,57]
Size exclusion chromatography	Based on the difference in the size of BEVs	~10^9^–10^10^ particles /μg protein	High structural integrity; high biological activity	Not suitable for large-scale separation; easily plugged gel	[59,60]
Protein precipitation	Based on the protein precipitation of BEVs in ammonium sulfate	~10^6^–10^8^ particles /μg protein	Simple process; low costs; suitable for large scale separation	Low efficiency; low purity	[61,62]
Affinity separation	Based on the specific binding between the targeted receptor of BEVs and the immobilized ligand of affinity chromatography	~10^6^–10^8^ particles /μg protein	Isolate exact subtypes of BEVs	Require additional separation; high costs	[63,64]

BEVs, bacterial extracellular vesicles

UC is one of the most commonly used isolation methods based on the difference in the density of BEVs [54]. It is simple to operate, but the yield of pure BEVs is relatively lower. To improve the purity of BEVs, DGC was developed based on the same principle, enabling the acquisition of a high yield of pure BEVs. However, it requires a longer time and higher cost to complete the operation [55]. By contrast, UF is the other most commonly used isolation method based on the difference in the size of BEVs [56,57]. It is simple to operate with the assistance of UF tubes. Yet, the specific pore size of the filter membrane makes it difficult to separate vesicles with a similar size, leading to the limited purity and yield of pure BEVs [58]. As such, SEC was developed based on the same principle [59]. Compared to UF, SEC can obtain BEVs with a higher yield of pure BEVs and structural and functional integrities [60]. However, it is only suitable for separating samples in small volumes and may result in clogging the gel columns at high risk. Since being enriched with proteins, BEVs can also be isolated using the protein precipitation method in ammonium sulfate solution [61]. This method is much simpler, less costly and suitable for large-scale separation, but the isolation efficiency and yield of pure BEVs are barely satisfactory [62]. Unlike the above methods, the affinity separation method isolates BEVs according to the specific binding between the targeted receptor of BEVs and the immobilized ligand of affinity chromatography [63]. This method only isolates the specific subtype of BEVs with high purity but low yield and requires additional separation and expensive cost [64].

**Fig. 3.**
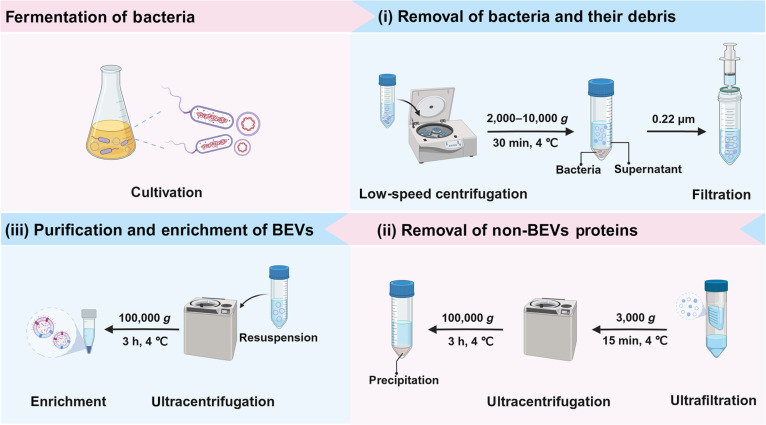
The combinational isolation procedure to obtain BEVs. After proper fermentation, BEVs can be collected following 3 main steps: (i) removal of bacteria and their debris by low-speed centrifugation and sterile filtration; (ii) removal of non-BEV proteins by ultrafiltration (UF) and ultracentrifugation (UC); and (iii) purification and extraction of BEVs by UC. All procedures are performed at 4 °C. The collected BEVs can be stored at −80 °C until further use. Created in BioRender.

In spite of the conventional isolation techniques developed, it is still difficult to extract BEVs at the desired yield and purity from the complex fermentation broths. Considering their own advantages and limitations, several of these isolation techniques were applied to obtain BEVs of higher quality. For instance, a combination of UC, UF, and DGC was created to collect the BEVs of *Akkermansia muciniphila* (AKK) from the fermentation medium rich in porcine mucin [65]. Notably, Ji et al. [66] have also proposed a combinational BEVs isolation protocol most recently, which is applicable to most bacteria. According to the protocol as illustrated in Fig. 3, the bacteria and their debris were firstly removed from the fermentation broth by 2,000 to 10,000×*g* low-speed centrifugation and the 0.22-μm sterile filter, respectively. Then, the non-BEV proteins were eliminated by the 50- to 100-kDa UF membrane together with 100,000×*g* UC. The preserved BEVs were finally purified and extracted by 100,000×*g* UC. Based on the proposed protocol, BEVs derived from various bacteria, including the gram-negative AKK and *Escherichia coli* Nissle 1917 (EcN), as well as the gram-positive *Lactobacillus rhamnosus GG* (LGG), have all been successfully obtained in high quality [67,68].

### Engineering strategies of BEVs

**Fig. 4.**
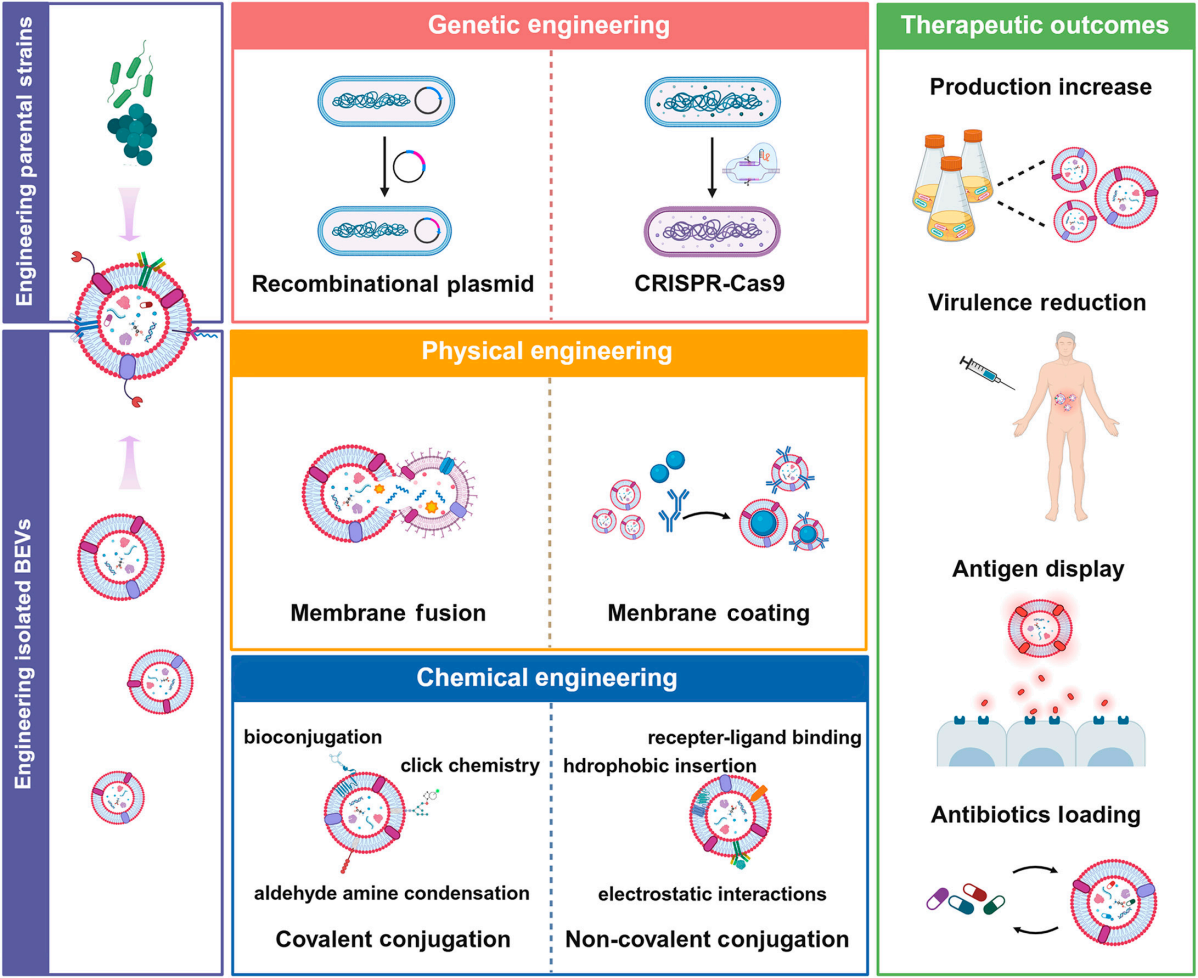
Engineering of BEVs for the optimal treatment of bacterial infections. These engineering strategies are categorized into two main approaches: engineering of the parental strains and engineering of the isolated BEVs. Engineering of the parental strains is achieved mainly through the recombinational plasmid-based and the CRISPR-Cas9-based gene overexpression or knock-out, which can lead to the virulence reduction, production increase, antigen presentation, and antibiotics loading of BEVs. Engineering of the isolated BEVs is achieved mainly through the membrane fusion and membrane coating-based physical engineering approaches, as well as the covalent conjugation and the noncovalent conjugation-based chemical engineering approaches, which can endow the isolated BEVs with additional capacities, including specific targeting, intracellular penetrating, antigen presentation, and antibiotics loading. Created in BioRender.

Even though the isolated BEVs have been used directly for the bacterial infection treatment, their therapeutic efficiencies are still hindered by several limitations, including the low yield, the high immunogenicity, and the insufficient antibacterial potency. To completely release the therapeutic potentials of BEVs, both of the parental strains and the isolated BEVs can be engineered with the advanced biological, physical, or chemical approaches (Fig. 4), generating BEVs with additional capacities to facilitate their optimal treatment for bacterial infections (Table 2).

**Table 2.** Engineering approaches of BEVs for the optimal treatment of bacterial infections: techniques, mechanisms, advantages, as well as limitations

Approaches	Techniques	Mechanisms	Advantages	Limitations	References
Genetic engineering	Recombinant plasmid-based engineering	Fusion of the specific gene sequences of the interested RNA/protein into recombinant plasmids	Mature and standardized manufacture	Limited cell type	[71,72,74–77]
	CRISPR-Cas9-based engineering	Specific cleavage or insertion of the gene sequences by CRISPR-Cas9	High specificity and precision	High costs; low production	[69,70]
Physical engineering	Membrane fusion	Rearrangement of phospholipid bilayer of BEVs with other lipid particles	High biocompatibility; high stability	Limited drug loading and release	[78,79]
	Membrane coating	Recombination of phospholipid bilayer of BEVs on the surface of nanoparticles	High biocompatibility; flexible functional allocation	Relatively low stability	[80,81]
Chemical engineering	Covalent conjugation	Formation of covalent chemical bonds in the processes of bioconjugation, aldehyde-amine condensation, and click chemistry	High stability; broad application	Residual organic reagent	[82,83,86]
	Noncovalent conjugation	Hydrophobic insertion of amphiphilic conjugates in the phospholipid bilayers of BEVs, electrostatic interactions between the charged molecules and BEVs, and the specific interactions between the paired biomolecules	Simple operation	Low stability	[84,85,87]

CRISPR-Cas9, Clustered Regularly Interspaced Short Palindromic Repeats-CRISPR associated protein 9; BEVs, bacterial extracellular vesicles

#### Engineering of parental strains

The engineering of parental strains is typically achieved through genetic engineering approaches, such as the recombinant plasmid-based and the CRISPR-Cas9-based gene overexpression or knockdown (Fig. 4) [69–71]. Recombinant plasmid-based engineering approaches have mature and standardized manufacturing, but are limited to the easily transfected cell types. CRISPR-Cas9-based engineering approaches perform outstandingly due to their high specificity and precision, while their high cost and low production still need more efforts to be well addressed.

Since the PAMP components carried by the BEVs, especially the LPS derived from gram-negative bacteria, can cause inflammatory responses of the host, these bioengineering approaches have been extensively used to reduce the biotoxicity of the BEVs [27]. Remodeling the key genes of LPS biosynthesis is the leading way. For instance, by knocking down the *lpxM* and *lpxL* genes of the lipid A, which is an important constituent of LPS, the immunogenicity of BEVs has been found to be markedly attenuated [72]. Besides, consecutive deletion of the genes that are responsible for encoding variant virulence factors in bacteria can also attenuate the potential toxicity of BEVs [73]. Such detoxification attenuation strategy is also applicable to BEVs derived from gram-positive bacteria. For instance, by knocking out the *agr* and *sea* genes that are responsible for the virulence factor expression in *S. aureus*, the pathogenicity of BEVs has also been found to be effectively eliminated [74].

Additionally, since the production of BEVs is related to the cross-linking degree between the bacterial membranes and the PGs, the fluidity of the bacterial membranes, as well as the condition of the bacterial growth, these bioengineering approaches can also be used to increase the production of the BEVs [75]. For instance, by knocking out the *pstA1* gene, the Pst/SenX3-RegX3 signaling pathway in *Mycobacterium tuberculosis* (*M. tuberculosis*) that is responsible for the production of BEVs could be activated [76]. By overexpressing the *PSMα* gene in *S. aureus*, the fluidity of the bacterial membranes has been found to increase with the mobilization of lipoproteins, thus promoting the release of the BEVs ultimately [77].

Moreover, functional proteins and peptides can be either fused with anchor motifs on the surface of bacteria or expressed inside the bacteria [17,29]. For instance, the *M. tuberculosis* antigens ESAT6, Ag85B, and Rv2660c have been attached to the surface of *E. coli* by fusion with the hemoglobin protease (Hbp), thus generating BEVs with multiple antigenic presentation [71]. Besides, the antibacterial peptide LL-37 has also been expressed inside the *E. coli*, thus generating BEVs with multiple antibacterial therapeutics.

#### Engineering of isolated BEVs

The engineering of isolated BEVs can be achieved through either a physical engineering or a chemical engineering approach. Since the compositions of BEVs secreted by the different bacteria or even the different strains of the same bacteria are heterogeneous, the membrane fusion of different BEVs can generate uniform BEVs with increased antigenic diversity [78,79]. Besides, liposomes have also been reported to coincubate with BEVs and thus confer additional functions of BEVs through membrane fusion [69]. The hybrid membrane vesicles fabricated by membrane fusion are biocompatible and stable, but might be limited by the relatively low drug loading and release efficiency. In comparison, the membrane coating refers to coating of the BEVs on the surface of antibacterial nanoparticles, including gold and silver nanoparticles, or antibiotic carrying nanoparticles, including the lipidic, polymeric, and inorganic nanoparticles. Besides, antibacterial molecules, such as antibacterial peptides, can also be loaded into BEVs with the assistance of either ultrasound or electroporation [80]. The homologous BEV coatings have been found to increase the targeting capacity of these antibacterial molecules or nanoparticles toward pathogens, facilitating the effective and safe treatment of bacterial infections [81]. Even though the complex vesicles fabricated by membrane coating are also biocompatible and have flexible functional allocation, they are not as stable as expected since the membrane of BEVs is transiently destroyed and reconstructed.

Apart from these physical engineering approaches, the isolated BEVs can also be modified through chemical engineering approaches. These approaches can be further divided into covalent conjugate modification, including bioconjugation, aldehyde-amine condensation, and click chemistry, and noncovalent conjugate modification, including hydrophobic insertion, electrostatic interaction, and receptor–ligand binding [82–86]. For instance, in combination with click chemistry and hydrophobic insertion, the BEVs isolated from LGG have been modified with the bone targeting peptide for controlled delivery [87]. Covalent conjugations endow BEVs with stable functional components for multiple applications, but residual organic reagents cannot be completely removed. Noncovalent conjugations are relatively easier to operate, but they usually lead to unstable conjugation. With these chemical engineering approaches, the surface of the isolated BEVs can be modified with additional functional motifs, such as targeting, penetrating, and antigen displaying peptides, empowering the optimal antibacterial efficiency of BEVs.

## Natural BEVs for Bacterial Infection Treatment

Because of the bioactive molecules they carried from their parental bacteria, the isolated natural BEVs can be exploited directly as antibacterial agents [88]. Besides, these natural BEVs with almost the same membrane compositions as their parental bacteria can also be exploited as antiadhesive agents [24]. To gain an in-depth understanding of their therapeutic potentials for bacterial infection treatments, recent investigations on these natural BEVs as both antibacterial and antiadhesion agents have been summarized below (Table 3), with emphasis on their antibacterial mechanisms.

**Table 3.** Natural BEVs exploited for the bacterial infection treatment: their strain sources, bioactive molecules, antibacterial mechanisms, and targeted bacteria

Strain sources	Bioactive molecules	Antibacterial mechanisms	Targeted bacteria	References
*P. aeruginosa*	Autolysin	Hydrolyze PG	*E. coli* DH5α, *E. coli* K-12, *P. vulgaris, S. flexneri, S. pullorum, S. arizonae, S. cholerae-suis, E. agglomerans, S. marcescens, K. pneumoniae*	[96]
*M. xanthus*	Proteases, phosphatases, and hydrolases	Damage membrane	*E. coli*	[100]
*M. xanthus*	Hydrolases and secondary metabolites	Damage membrane	*E. coli*	[97]
*B. thailandensis*	Hydrolases, HMNQ, and rhamnolipid	Hydrolyze PG, disrupt proton motility, inhibit pyrimidine synthesis, limit biofilm generation, and compete for an environmental niche	MRSA, *S. aureus*, *A. baumannii*, MDR *A. baumannii*, MDR *C. albicans*	[101]
*Cystobacter* sp. *Cbv34*	Cystobactamids	Specifically inhibit bacterial topoisomerase	*E. coli*, intracellular MRSA	[23]
*B. subtilis*	Phage attachment molecules	Resistant-sensitive transformation	*B. subtilis*	[106]
*H. pylori*	Adhesins	Competitively inhibit the parent bacteria’s adhesion	*H. pylori*	[112]
*L. crispatus* BC5 and *L. gasseri* BC12	Adhesins	Competitively inhibit the parent bacteria’s adhesion	*E. coli, S. aureus, S. agalactiae*, and *E. faecalis*	[113]

PG, peptidoglycan; HMNQ, 4-hydroxy-3-methyl-2-(2-nonenyl)-quinoline; MRSA, methicillin-resistant *Staphylococcus aureus*; MDR, multidrug resistant; *P. aeruginosa*, *Pseudomonas aeruginosa*; *E. coli*, *Escherichia coli*; *P. vulgaris*, *Proteus vulgaris*; *S. flexneri*, *Shigella flexneri*; *S. pullorum*, *Salmonella pullorum*; *S. arizonae*, *Salmonella arizonae*; *S. cholerae-suis*, *Salmonella cholerae-suis*; *E. agglomerans*, *Enterobacter agglomerans*; *S. marcescens*, *Serratia marcescens*; *K. pneumoniae*, *Klebsiella pneumoniae*; *M. xanthus*, *Myxococcus xanthus*; *B. thailandensis*, *Burkholderia thailandensis*; *S. aureus*, *Staphylococcus aureus*; *A. baumannii*, *Acinetobacter baumannii*; *C. albicans*, *Candida albicans*; *B. subtilis*, *Bacillus subtilis*; *H. pylori*, *Helicobacter pylori*; *L. crispatus*, *Lactobacillus crispatus*; *L. gasseri*, *Lactobacillus gasseri*; *S. agalactiae*, *Streptococcus agalactiae*; *E. faecalis*, *Enterococcus faecalis*

### BEVs as antibacterial agents

**Fig. 5.**
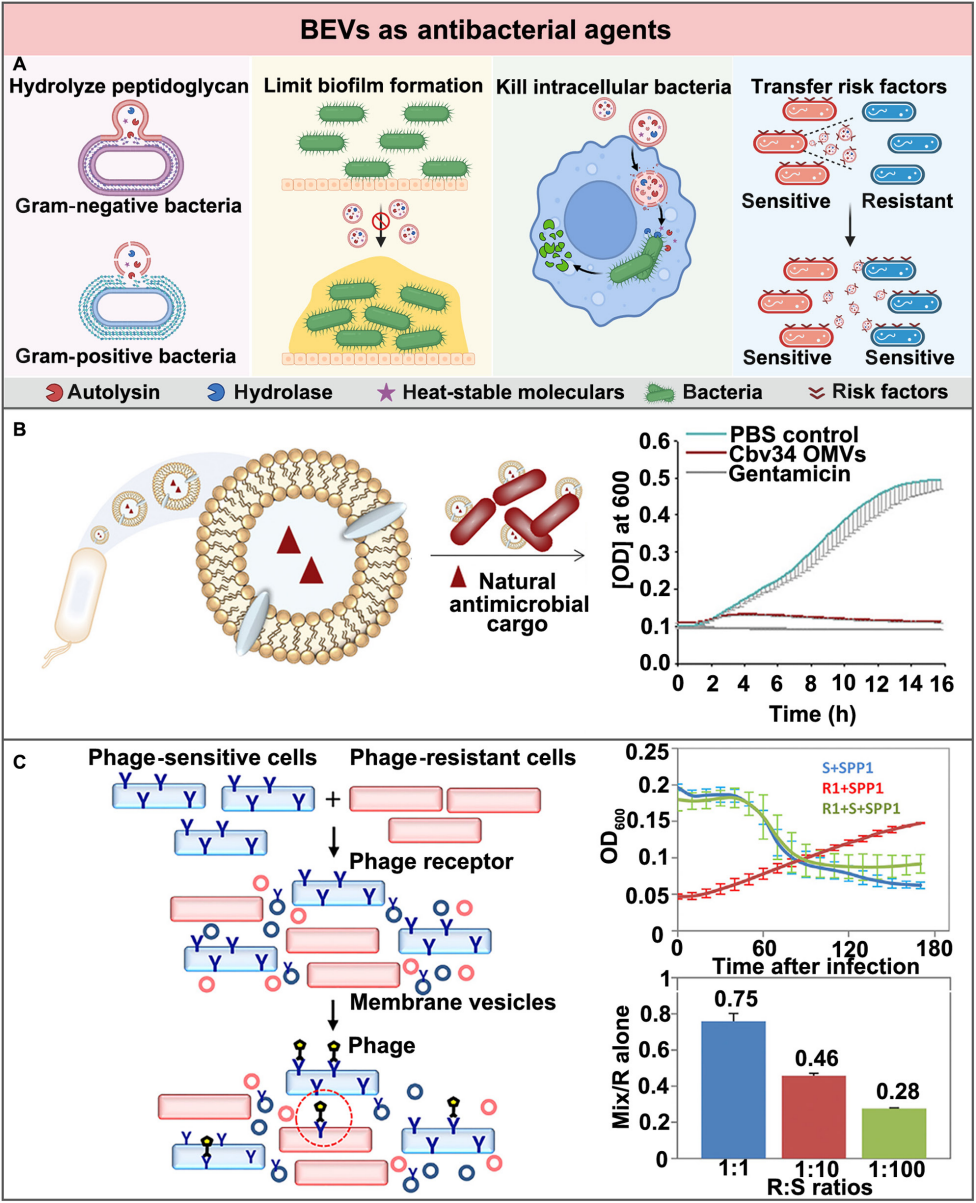
BEVs as antibacterial agents for bacterial infection treatment. (A) Schematic illustration of the antibacterial mechanisms of BEVs as antibacterial agents. The natural BEVs containing the antibacterial components such as autolysin, hydrolase, and heat-stable molecules could lyse bacteria, limit biofilm formation, kill intracellular bacteria, and enhance susceptibility to bacterial infection treatment. Created in BioRender. (B) The myxobacterium *Cystobacter* sp. Cbv34-derived BEVs exhibited a more obvious killing effect against the intracellular MRSA than the commercialized antibiotic gentamicin. Adapted from Ref. [23] with permission. Copyright 2018, Elsevier BV. (C) The phage-sensitive *B. subtilis-*derived BEVs could transmit their phage attachment molecules to the phage-resistant *B. subtilis*, leading to their infection and lysis by phage. Adapted from Ref. [106] with permission. Copyright 2017, Elsevier Inc.

To gain a competitive advantage in survival, most bacteria have evolved with antibacterial capacities to suppress their competing bacteria [14]. These capacities are achieved primarily through endogenous bioactive substances, such as autolysin, hydrolase, and thermostable antibacterial molecules [88–90]. The secretion of BEVs is an important way for bacteria to release these bioactive substances. The secreted BEVs can provide protection and enable delivery of these bioactive substances into competing bacteria, subsequently inhibiting the growth of competing bacteria [23]. Meanwhile, the secreted BEVs can carry these bioactive molecules to inhibit or eradicate biofilms as well [91,92]. Besides, the BEVs with the lipid bilayer nanostructure have been exploited to penetrate host cells, killing the pathogenic bacteria intracellularly [93]. Moreover, apart from these direct antibacterial activities, the secreted BEVs also possess indirect antibacterial effects, delivering sensitizers to facilitate the antibacterial process (Fig. 5A).

Hydrolases that are capable of lysing bacteria were the first antibacterial substances identified from BEVs. These BEVs containing the hydrolytic autolysin were found to be produced by *P. aeruginosa* in 1996 [88]. Since their discovery, BEVs with the autolysin have been extensively studied due to their antibacterial potential. As reported, autolysin is an endogenous lytic enzyme that widely exists in bacterial cells. It can hydrolyze PG, which is one of the most important components of the bacterial cell wall to maintain intracellular osmotic pressure [94]. Both gram-positive and gram-negative bacteria could transport autolysins via BEVs to other bacteria, thus causing their disintegration [95–97]. Furthermore, the chemical property and the targeting specificity of the autolysin in BEVs have also been investigated. For instance, Watt and Clarke [98] reported that the autolysin derived from the *P. aeruginosa* PAO1 was a 26-kDa β-glycosidase, and it was released in the BEV encapsulated form rather than in the free form. Besides, they found that the autolysin in the BEV encapsulated form has higher activity against the bacteria with a PG structure similar to the parent bacteria, compared to the parent bacteria or the bacteria with a different PG structure [99]. Apart from autolysin, the abundant hydrolases in BEVs derived from the predatory bacteria have also attracted increasing attention. For instance, as a soil predatory bacterium, *Myxococcus xanthus* (*M. xanthus*) contains various hydrolases to help it prey and digest nutrients. Besides, a number of secondary metabolites in *M. xanthus* were also identified to be beneficial for their bactericidal activity [100]. Therefore, the BEVs derived from *M. xanthus* have been found to possess these hydrolases and secondary metabolites and thus exert antibacterial activity against *E. coli* [97].

Thermostable small molecules that can inhibit or eradicate biofilm have also been identified in BEVs. For instance, Wang et al. [101] found that BEVs derived from the heat-inactivated *Burkholderia thailandensis* (*B. thailandensis*) still exhibited a markedly inhibitory effect on the growth of *S. aureus*. However, autolysin and other hydrolases are thermally labile. Subsequently, the thermostable rhamnolipids and 4-hydroxy-3-methyl-2-(2-nonenyl)-quinoline (HMNQ) with antibacterial activities were identified. Rhamnolipids are 3-hydroxyfatty acids linked by single or double rhamnose through a β-glycosidic bond [102]. As biosurfactants, the antibacterial and antibiofilm capacities of rhamnolipids have been previously reported [103–105], while HMNQ was firstly found to effectively suppress the biofilm preformation of the methicillin-resistant *S. aureus* (MRSA) in the study. Owing to the coexistence of both hydrolase and thermostable small molecules, the BEVs derived from *B. thailandensis* exhibited considerable antibacterial activity against a broad spectrum of bacteria, including gram-positive bacteria, gram-negative bacteria, and even the multidrug-resistant (MDR) bacteria.

In addition to their capacities against planktonic bacteria and bacteria in biofilms, BEVs have also been found to possess capacity against intracellular bacteria. Owing to their lipid bilayer nanostructures that are similar to mammalian cells, BEVs can enter host cells and kill intracellular pathogenic bacteria effectively. As demonstrated in Fig. 5B, the intracellular bacterial killing capacities of BEVs were validated on MRSA-infected cells [23]. Compared to the free form gentamicin and cystobactamids, the myxobacterium *Cystobacter* sp. Cbv34-derived BEVs containing cystobactamids have exhibited a more obvious killing effect against the intracellular MRSA.

Moreover, BEVs can also integrate the specific antigens from the parent bacteria into the outer membrane of the target bacteria to achieve bactericidal effects. After the antigen integration by BEVs, the survival risk of the target bacteria being identified and cleared markedly increased; thus, the killing effect should be enhanced accordingly. The hypothesis on these resistant-sensitive transformations was subsequently confirmed by Tzipilevich et al. [106]. As demonstrated in Fig. 5C, through the BEV-mediated transformation, the phage attachment molecules derived from the phage-sensitive *Bacillus subtilis* (*B. subtilis*) could be integrated into the phage-resistant *B. subtilis*. Subsequently, even the phage-resistant *B. subtilis* could be effectively infected and lysed by phage.

In conclusion, natural BEVs possess inherent antibacterial capacities owing to their endogenous bioactive molecules. They can function as antibacterial agents that directly eliminate competing bacteria, disrupt biofilm, or sensitize resistant bacteria via membrane antigen integration. However, these natural BEVs usually lack targeting activities toward the specific strain and can hardly distinguish between the pathogenic bacteria and the host’s beneficial commensal microbiota. In the future, all genetic, chemical, and physical engineering approaches could be ingeniously utilized to modify the surface of these natural BEVs with targeting molecules like peptides and circumvent their off-target effects and potential toxicities during bacterial infection treatment.

### BEVs as antiadhesion agents

**Fig. 6.**
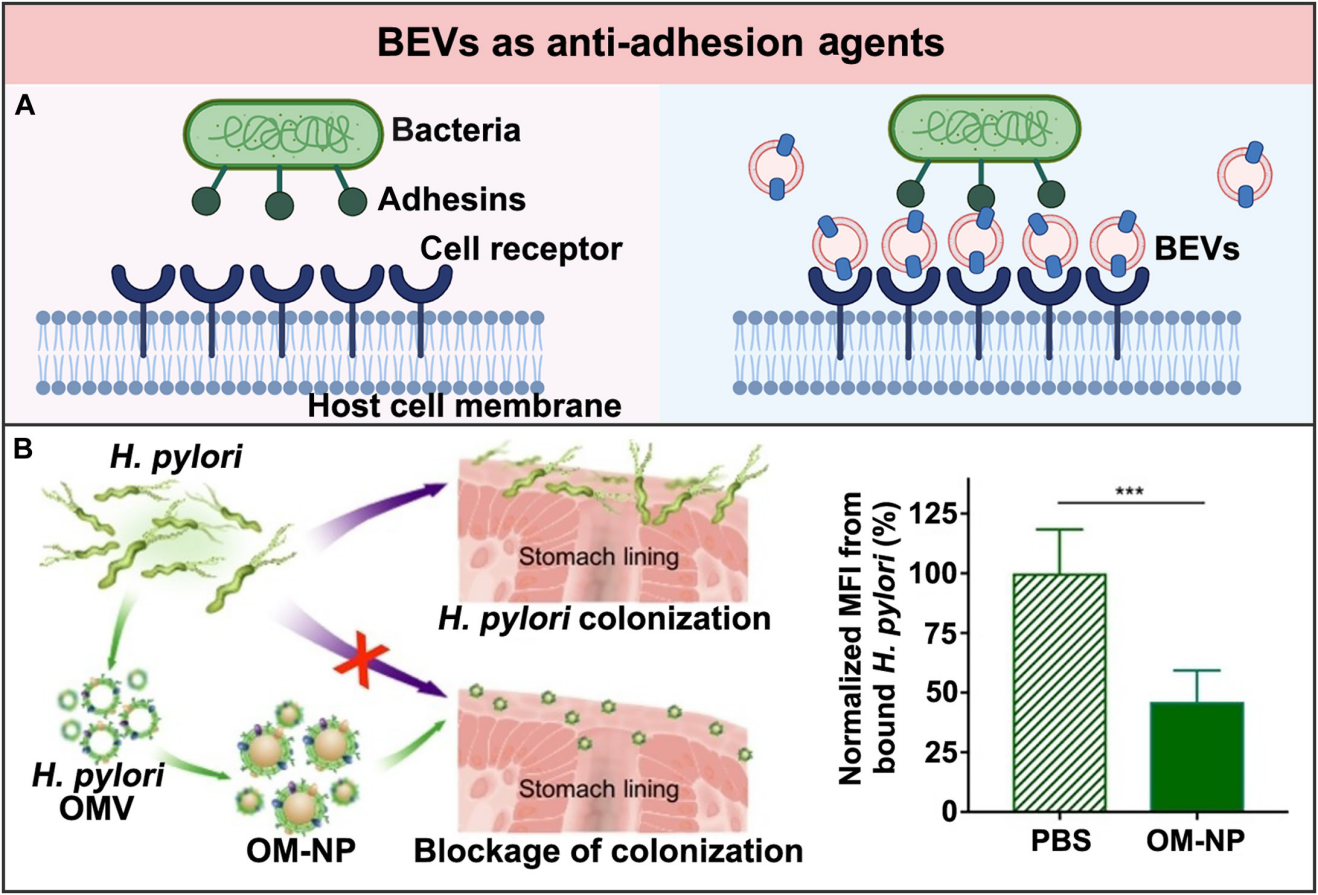
BEVs as antiadhesion agents for bacterial infection treatment. (A) Schematic illustration of the antibacterial mechanisms of BEVs as antiadhesion agents. Created in BioRender. (B) The *H. pylori*-derived BEVs could attach to the stomach lining, competitively inhibiting the adhesion and colonization of *H. pylori* on the stomach lining. Adapted from Ref. [112] with permission. Copyright 2019, Wiley-VCH.

Adhesion to the host cells and tissues is the first step for bacterial infection [107,108]. Through recognition and binding to adhesin receptors on the surface of cells or tissues, adhesion can be achieved for bacteria, facilitating their further colonization and infection (Fig. 6A) [109–111]. As such, both adhesin analogs and receptor analogs have been developed as antiadhesion agents, blocking the interactions between bacterial adhesins and host receptors. Since the bacterial growth cycle is not affected, antiadhesion therapy has been considered as an effective strategy to prevent and treat bacterial infections without the occurrence of antibiotic resistance.

BEVs that inherit adhesins from their parental bacteria have been intensively exploited as adhesin analogs to competitively inhibit the adhesion and infection of the pathogenic bacteria to their host cells. For instance, Zhang et al. [112] have used the *H. pylori-*derived BEVs to competitively adhere to the gastric mucosa, thereby effectively inhibiting the adhesion and colonization of *H. pylori* in the stomach (Fig. 6B). Similarly, Liu et al. [25] have used the *H. pylori-*derived BEVs to fabricate hybrid vehicles for the prevention and treatment of *H. pylori* infection. Besides, Croatti et al. [113] have demonstrated that the symbiotic *Lactobacillus*-derived BEVs could markedly reduce the adhesion of all examined pathogens to hosts because the abundant amounts of adhesins on BEVs have saturated all the adhesin receptors presented by hosts.

In conclusion, natural BEVs intrinsically possess antiadhesion capacities owing to surface adhesins inherited from their parental bacteria. They can function as either adhesin analogs or receptor analogs, competitively blocking the interactions between bacterial adhesins and host receptors and thus preventing bacterial infections. Nonetheless, these natural BEVs can only be adhesive to bacterial strains with the same adhesins, while they have limited adhesion and retention on the mucosa. In the future, these natural BEVs could be engineered to enhance their targeted delivery, mucosal retention, and broad-spectrum inhibition, amplifying their therapeutic effectiveness for the bacterial infection treatment.

## Engineered BEVs for Bacterial Infection Treatments

Even though the naturally secreted BEVs can be used directly as antibacterial or antiadhesion agents, the emerging advanced engineering approaches can further endow natural BEVs with additional capacities to broaden their roles for bacterial infection treatment. For instance, based on the different engineering approaches, BEV-based vaccines can be fabricated with the appropriate and adequate presentation of both endogenous or exogenous antigens for bacterial infection treatment [114,115]. Besides, based on the different engineering approaches, the BEV-based nano-delivery system can also be constructed with the targeted delivery of both endogenous or exogenous antibacterial agents for bacterial infection treatment [116–118]. All these engineered BEVs with anti-infective capacities have been summarized and discussed below (Table 4).

**Table 4.** Engineered BEVs exploited for the bacterial infection treatment: their anti-infective roles, strain sources, bioactive cargoes, engineering approaches, and anti-infective applications

Anti-infective roles	Strain sources	Bioactive cargoes	Engineering approaches	Administration routes	Anti-infective applications	References
Antigen vaccines	*N. meningitidis*	–	Removal of lipoxins by detergent DOC	–	Attenuated vaccine against *N. meningitidis* group B	[131]
	*E. coli* O157:H7	–	Mutational inactivation of the MsbB lipid A acyltransferase	–	Attenuated vaccine against *E. coli*	[132]
	*N. meningitidis*	fHbp antigen	Gene copy integration	Intramuscular injection	Trivalent vaccine against *N. meningitidis*	[136]
	*N. meningitidis*	PorA	Gene cloning and multicopy integration	Subcutaneous injection	Multivalent vaccine against *N. meningitidis*	[138]
	*S. flexneri*	–	Encapsulation of poly(anhydride) nanoparticles into the BEVs by solvent displacement	Nasal or oral administration	Vaccine against *S. flexneri*	[142]
	*E. coli*	–	Encapsulation of gold nanoparticles into BEVs by extrusion	Subcutaneous injection	Vaccine against *E. coli*	[143]
	*B. bronchiseptica*	–	Encapsulation of GA nanoparticles into BEVs by extrusion	Subcutaneous injection	Vaccine against *B. bronchiseptica*	[144]
	*E. coli*	NMVs	Encapsulation of lipid nanoparticles into BEVs by extrusion	Subcutaneous injection	Vaccine against *E. coli*	[168]
	*P. aeruginosa*	–	Nitrogen cavitation	Intraperitoneal injection	Vaccine against *P. aeruginosa*	[145]
Vaccine adjuvants	*N. lactamica*, *N. meningitidis*	HBsAg	Mixing	Subcutaneous injection, intraperitoneal injection, or intranasal administration	Vaccine adjuvant against *N. meningitidis*	[148]
	*E. coli* W3110	KLH, SIINFEKL peptide	Mixing	Subcutaneous injection	Vaccine adjuvant against model peptide antigen	[149]
	*E. coli*	GFP	Mixing, engineering via ClyA fusion protein transporter platform	–	Vaccine adjuvant against model subunit antigen GFP	[150]
	*N. meningitidis* group B M986 NCV1	BPS	Covalently conjugation	–	Vaccine adjuvant against *N. meningitidis* group B	[151]
	*N. meningitidis*	LPS of *B. abortus* S99	Covalent conjugation	Subcutaneous injection	Vaccine adjuvant against brucellosis	[152]
	*E. coli*	SpyTag-linked to malaria antigens (CIDR and Pfs25)	Covalent conjugation with VLPs that are genetically expressed SpyCatcher by mixing	Intramuscular injection	Vaccine adjuvant against malaria	[153]
	*E. coli*	GPI-anchored tdTomato (RFP) and GFP	Lipid anchoring	–	Vaccine adjuvant against model subunit antigen RFP and GFP	[154]
	*E. coli, S. enterica*	*M. tuberculosis* antigens (ESAT6, Ag85B, and Rv2660c), epitopes from *C. trachomatis* MOMP	Hbp autotransporter platform	–	Vaccine adjuvant against *M. tuberculosis*	[71]
	*E. coli*	GFP	ClyA fusion protein transporter platform	Subdermal ear injection	Vaccine adjuvant against model subunit antigen GFP	[155]
	*E. coli*	*A. baumannii* outer membrane protein Omp22	ClyA fusion protein transporter platform	Subcutaneous injection	Vaccine adjuvant against *A. baumannii*	[156]
	*E. coli*	GFP	Fusion with Tat signal sequence	–	Vaccine adjuvant against model subunit antigen GFP	[158]
	*E. coli*	HtrA	Fusion with OmpA sequence	Intramuscular injection	Vaccine adjuvant against *C. muridarum*	[159]
	*E. coli* Nissle 1917	Antigens of SARS-CoV-2 NG06 and RBD	Fusion with ClyA and OmpA sequences	Intraperitoneal injection	Vaccine adjuvant against SARS-CoV-2	[160]
	*E. coli*	*S. pneumoniae* CPS14, *C. jejuni* heptasaccharide N-glycan	Genetic expression by plasmid transfection	Intraperitoneal injection	Vaccine adjuvant against *S. pneumoniae* and *C. jejuni*	[161]
	*E. coli*	O-PS from *F. tularensis*	Genetic expression by plasmid transfection	Intraperitoneal injection	Vaccine adjuvant against *F. tularensis*	[162]
Antibiotic carriers	*P. aeruginosa*	Gentamicin	Coincubation	–	Antibiotic delivery	[88,167]
	*A. baumannii*	Ceftriaxone, amikacin, azithromycin, ampicillin, levofloxacin, ciprofloxacin, and norfloxacin	Coincubation	Oral administration	Antibiotic delivery	[116]
	*E. coli*	Rifampicin, MSNs	Encapsulation of MSNs and rifampicin into BEVs by probe ultrasonication	Intraperitoneal injection	Target delivery of antibiotic to *E. coli*	[170]
	*E. coli*	Norfloxacin, NMVs	Fusion with neutrophil membrane vesicles; encapsulation of lipid nanoparticles into BEVs by extrusion	Intravenous injection or intratracheal administration	Target delivery of antibiotic to the inflammatory site	[168]

HBsAg, hepatitis B virus surface antigen; KLH, keyhole limpet hemocyanin; GFP, green fluorescent protein; BPS, *Neisseria meningitidis* group B capsular polysaccharide; LPS, lipopolysaccharide; GPI, glycosylphosphatidylinositol; RFP, red fluorescent protein; Tat, twin-arginine; Hbp, hemoglobin protease; MOMP, major outer membrane protein; RBD, receptor-binding domain; CPS14, serotype 14 capsule; O-PS, O-antigen polysaccharides; DOC, deoxycholate; PorA, porine A; BSA, bovine serum albumin; GA, glycyrrhizic acid; NMVs, neutrophil membrane vesicles; MSNs, mesoporous silica nanoparticles; *N. meningitidis*, *Neisseria meningitidis*; *E. coli*, *Escherichia coli*; *S. flexneri*, *Shigella flexneri*; *B. bronchiseptica*, *Bordetella bronchiseptica*; *P. aeruginosa*, *Pseudomonas aeruginosa*; *N. lactamica*, *Neisseria lactamica*; *B. abortus*, *Brucella abortus*; *S. enterica*, *Salmonella enterica*; *M. tuberculosis*, *Mycobacterium tuberculosis*; *C. trachomatis*, *Chlamydia trachomatis*; *A. baumannii*, *Acinetobacter baumannii*; *C. muridarum*, *Chlamydia muridarum*; *S. pneumoniae*, *Streptococcus pneumoniae*; *C. jejuni*, *Campylobacter jejuni*; *F. tularensis*, *Francisella tularensis*; *B. subtilis*, *Bacillus subtilis*

### BEVs as antigens in vaccination

**Fig. 7.**
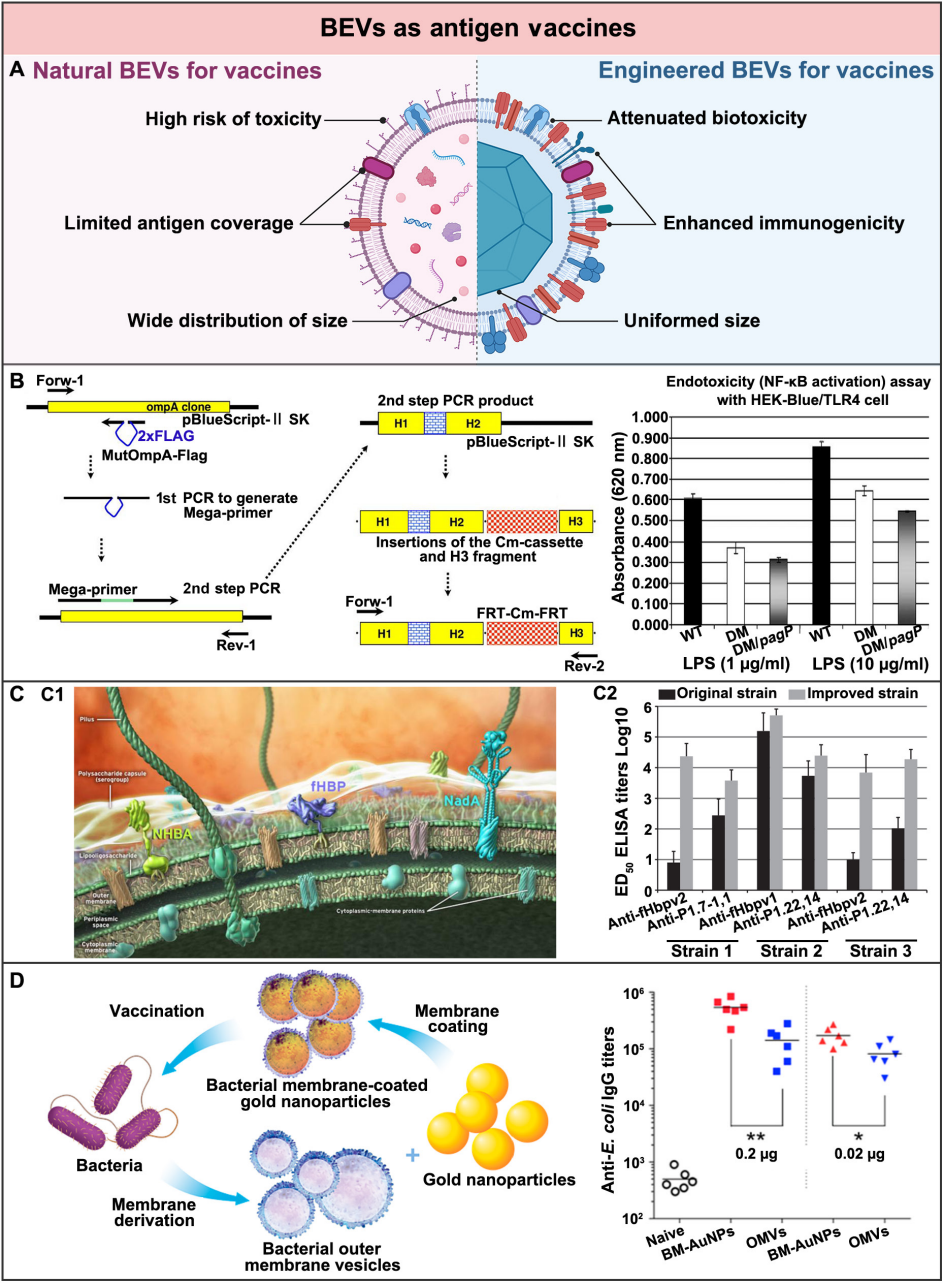
BEVs as antigens in vaccination for bacterial infection treatment. (A) Schematic illustration of the comparison of the natural and engineered BEVs as antigens for vaccination. Created in BioRender. (B) By genetic engineering, the lipid A segment of LPS on the *E. coli*-derived BEVs was adjusted, generating BEV vaccines with attenuated endotoxicity but preserved immunogenicity. Adapted from Ref. [132] with permission. Copyright 2009, Elsevier BV. (C) (C1) Schematic representation of the fHbp antigens displayed at the surface of the *N. meningitidis.* Adapted from Ref. [135] with permission. Copyright 2012, Elsevier Ltd. (C2) By genetically inserting an additional fHbp gene into the porB locus of *N. meningitidis*, a trivalent BEV vaccine that could elicit potent antibody responses against *N. meningitidis* infection was generated. Adapted from Ref. [136] under the Creative Commons CC-BY-NC-ND. (D) *E. coli*-derived BEVs were coated onto AuNPs to obtain vaccines with enhanced stability and potency, eliciting bacterium-specific immunity against *E. coli*. Adapted from Ref. [143] with permission. Copyright 2015, American Chemical Society.

Vaccination is one of the most effective ways to induce protective immunity and prevent bacterial infection. By mimicking bacterial pathogens, vaccines can stimulate both innate and adaptive immunity to prevent infection but do not cause disease [119]. These imply that the fully functionalized vaccines should possess PAMPs, pathogen-specific antigens, and appropriate sizes [120]. As such, BEVs with inherent structures and components similar to their parent strains have been considered as natural vaccines. The PAMPs and pathogen-specific antigens displayed at their surface can activate the adaptive immunity and enhance the innate immunity [121]. Owing to advanced engineering techniques, the potential limitations of the natural BEVs being used directly as antigenic vaccines, such as the high LPS-associated toxicity, the limited antigen-spectrum coverage, and the heterogeneous size distribution, have also been overcome, making these BEVs attractive alternatives for novel vaccine formulations (Fig. 7A).

Compared to conventional bacterial vaccines, natural BEV vaccines have attracted increasing interests. Typically, conventional bacterial vaccines are produced following the isolation–inactivation–injection procedure developed from the attenuated strains [122]. Since BEVs cannot replicate and require no inactivation, they are easier to produce and safer to use. The earliest BEV vaccines were made from *Neisseria meningitidis* (*N. meningitidis*) group B. These vaccines have successfully fought outbreaks of *N. meningitidis* group B in Cuba, Norway, Chile, and New Zealand [123–126]. By far, these vaccines are still the only commercialized BEV products that have been used for clinical treatment.

Since then, BEVs derived from other pathogenic bacteria have also been used as vaccines against diverse bacterial infections. For instance, the *P. aeruginosa*-derived BEV vaccine has been reported to protect the mouse with acute lung infection that is lethally challenged by *P. aeruginosa* [127]. Besides, *Burkholderia pseudomallei* (*B. pseudomallei*)-derived BEVs have also been demonstrated to effectively protect melioidosis mice that were lethally challenged by the *B. pseudomallei* aerosol [128].*B. pseudomallei*-derived BEVs have been further found to provide cross-protection against mice and even nonhuman primates infected with the *Burkholderia mallei* (*B. mallei*) aerosol [129]. These cross-protections have been achieved based on the fact that the most virulent determinants are highly conserved between *B. pseudomallei* and the *B. mallei*. Nonetheless, the direct vaccination of these natural BEVs for bacterial infection treatment still faces a lot of challenges, such as potential toxicity, insufficient immunity, and heterogeneous efficiency.

The PAMPs carried by the natural BEVs, including LPS and other types of virulence, not only can activate the immune responses but also may cause side effects [130]. Therefore, different engineering approaches have been used to optimize the immunogenicity of the BEV vaccines, so that the moderate immunostimulatory capacity of these BEV vaccines can be preserved with reduced biotoxicity. Among all these engineering approaches, removal of the nonspecific antigens presented by BEVs is an effective strategy. For instance, Holst et al. [131] firstly used detergents to remove lipoxins from gram-negative bacteria, so that the attenuated vaccines of their BEVs were produced. However, with the decreasing LPS presentation and subsequent biotoxicity, the immunostimulatory effectiveness of these BEV vaccines decreased as well. As such, nondetergent strategies based on genetic detoxification have gained more and more interest. For instance, van der Ley et al. and Kim et al. [72,132] have used the genetic engineering approach to alter the lipid A segment of LPS from full endotoxin hexa-acylation to penta-acylation. The attenuated *E. coli*-derived BEVs were demonstrated to still preserve adequate immunostimulatory activities (Fig. 7B) [132]. Through genetic engineering of LPS, more and more attenuated BEV vaccines generated by various bacteria have been reported, such as *N. meningitidis* [133] and *Vibrio cholerae* [134]. These attenuated BEV vaccines with reduced biotoxicity but sufficient immunogenicity show promise as the next generation of bacterial vaccines.

Besides, the specific antigens presented by the natural BEVs are usually too low to induce effective antibody titers in the host [121]. Therefore, genetic engineering approaches have also been used to increase the expression level of antigens on BEV vaccines. For instance, Zhang et al. [135,136] have added an fHbp gene copy into the porB locus of *N. meningitidis* to increase the expression level of fHbp antigen. As a result, the immunogenicity of the generated trivalent BEV vaccine improved, inducing a remarkable antibody titer in the host to fight against bacterial infection (Fig. 7C). In addition, multivalent BEV vaccines have also been genetically engineered, considering that there is usually more than one serotype of antigens in the same pathogenic bacteria [137]. For instance, Claassen et al. [138] have genetically engineered *N. meningitidis* to generate a BEV vaccine displaying multivalent porine A (PorA). In a further phase I clinical study, about half of the volunteers who received the BEV vaccine with 6 PorA subtypes were found to have at least a 4-fold increase in the expression of the antimicrobial antibodies [139]. These engineered BEV vaccines with improved expression level and diversity of specific antigens are believed to exert enhanced potency against bacterial infections effectively.

Moreover, the size heterogeneity of natural BEVs is also considered to affect their immunostimulatory effects. Typically, BEV vaccines with a particle size between 20 and 100 nm mainly enter the lymph nodes through lymphatic drainage, while BEV vaccines with a larger particle size (less than 500 nm) are preferentially absorbed and delivered by antigen-presenting cells (APCs) to the lymph nodes [140]. By contrast, BEV vaccines with particle sizes larger than 500 nm are usually phagocytosed by macrophages. Apart from those derived from the different strains of bacteria, the size distribution differences even exist in the BEVs derived from the same bacteria [141]. Therefore, different engineering approaches have been used as well to control the size of BEV vaccines. For instance, Camacho et al. [142] have encapsulated poly(anhydride) nanoparticles into *Shigella* derived BEVs, generating the BEV-based vaccines with a uniform size of 148 nm. Compared to *Shigella*-derived BEVs, a lower dose of the engineered BEVs was needed to stimulate the same immune response. Similarly, Gao et al. have loaded 30-nm gold nanoparticles (AuNPs) into *E. coli*-derived BEVs. The generated BEV vaccines not only possessed enhanced stability but also exhibited outstanding potency to induce higher and longer antibody titers (Fig. 7D) [143]. Lately, both bovine serum albumin and glycyrrhizic acid nanoparticles, as well as lipid nanoparticles, have also been used to construct homogeneous BEV vaccines to achieve enhanced antibody titers for effective bacterial infection treatment [144]. In addition to the assistance from nanoparticles, advanced fabrication techniques have also been exploited to control the size of BEV vaccines. For instance, Wang et al. [145] have used nitrogen cavitation to rapidly form the nano-vesicular vaccine consisting of a double-layered membrane derived from BEVs. The engineered BEVs exhibited a narrowly distributed size but widely expressed antigens, stimulating an effective immune response to improve the survival rate of sepsis mice infected by *P. aeruginosa*. These engineered BEV vaccines with controlled size offer more possibilities to adjust their immune effectiveness for optimal bacterial infection treatment.

In conclusion, the PAMPs and strain-specific antigens of BEVs formed the foundation for their applications as antigen vaccines against bacterial infections. Limitations, such as the high LPS-associated toxicity, the limited antigen-spectrum coverage, and the heterogeneous size distribution, could be overcome in the future through deliberate design and engineering strategies. Key engineering approaches include genetic detoxification, genetic antigen enhancement, and nanoparticle coating, which could be employed accordingly. These strategies transform the natural BEVs into the next generation of engineered vaccine platforms with enhanced safety, antigenicity, and immunogenic efficacy for preventing and treating bacterial infections.

### BEVs as adjuvants in vaccination

Compared to the commonly used vaccine adjuvants, including alum, cholera toxin, and diphtheria toxin, that have exhibited some adverse side effects like toxicity, inflammation, and less effective mucosal immunity, bacteria-derived BEVs are promising candidates with several superiorities similar to the vaccine adjuvants. First of all, bacteria-derived BEVs contain a series of complex PAMPs, such as LPS, PG, and flagellin [146]. These inherent PAMPs can endow BEVs with the ability to induce the maturation of APCs, promoting antigen presentation and thus enhancing immune responses [147]. Besides, the nanoscale structure of BEVs can facilitate their transport into lymph nodes through lymphatic drainage or after being phagocytosed by APCs to activate the immune system [140]. Moreover, emerging genetic engineering techniques can functionalize the parent bacteria, thus generating BEVs expressing heterologous antigens to achieve the desired immunogenicity [71].

**Fig. 8.**
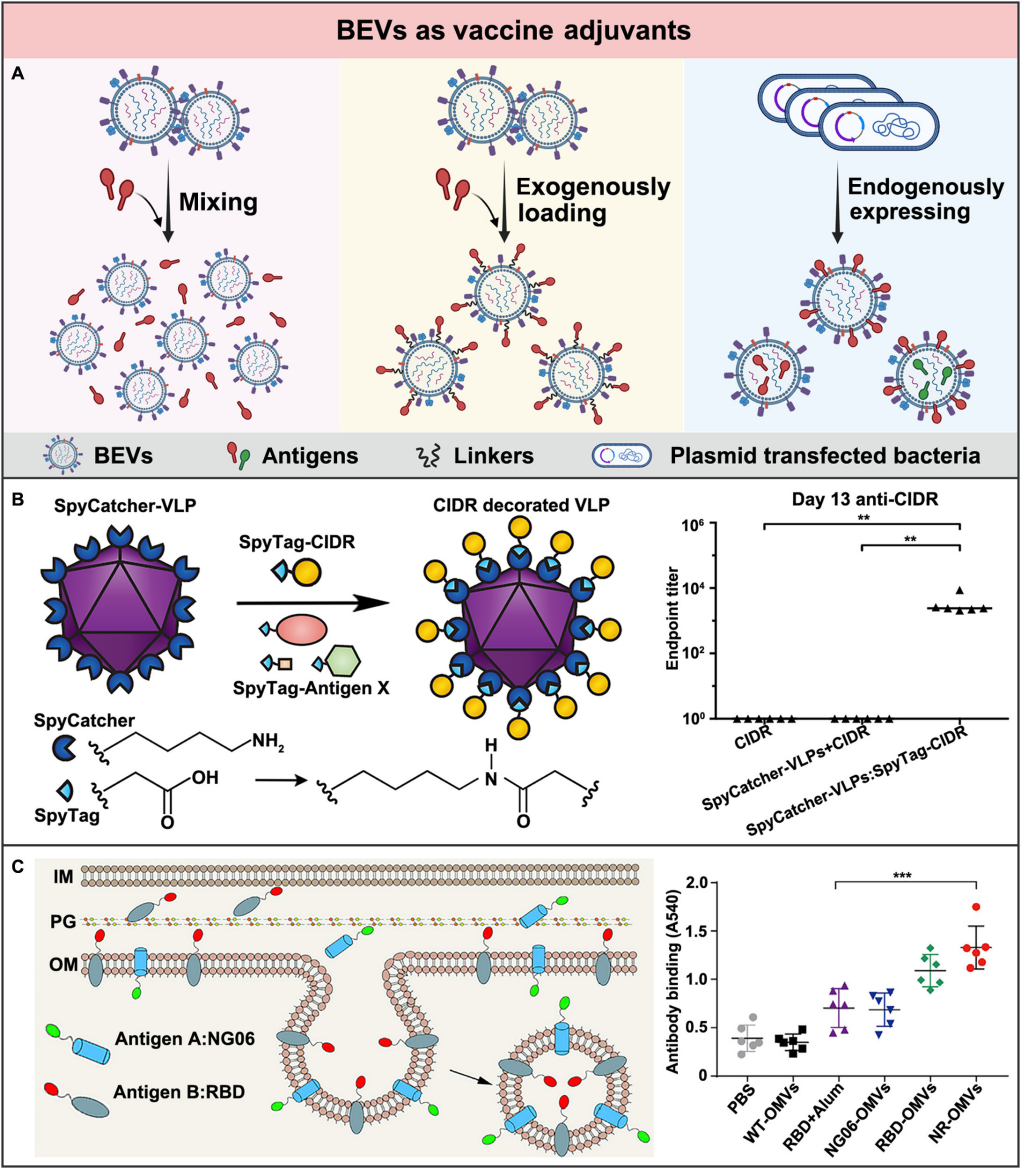
BEVs as adjuvants in vaccination for bacterial infection treatment. (A) Schematic illustration of the construction mechanisms of BEVs as adjuvants for the antigen vaccines, including mixing, exogenously loading, and endogenously expressing. Created in BioRender. (B) Based on the SpyCatcher and SpyTag platform, the BEVs adjuvanted with the exogenous malaria antigens are chemically conjugated at their surface, markedly protecting mice from malaria. Adapted from Ref. [153] under the Creative Commons Attribution 4.0 International License. (C) Based on the ClyA and OmpA membrane fusion proteins, the BEVs adjuvanted with the endogenous antigens being expressed both at their surface and in their lumen markedly enhanced the immunity against SARS-CoV-2. Adapted from Ref. [160] under the Creative Commons CC BY-NC-ND 4.0.

With excellent immune activation capacity, BEV adjuvants can be combined with protein-based vaccines by mixing, exogenous loading, or endogenous expression (Fig. 8A). The adjuvant capability of BEVs was initially found by simply mixing them with pathogenic antigens. As such, BEVs could noncovalently bind to antigens through electrostatic interaction, hydrophobic interaction, or hydrogen bonding. Compared to vaccination with the hepatitis B virus surface antigen (HBsAg) alone, coimmunization of the HBsAg together with either *Neisseria lactamica* or *N. meningitidis*-derived BEVs achieved higher specific immunoglobulin G (IgG) levels [148]. These results suggested the remarkable adjuvant activities of these BEVs for the recombinant HBsAg. Similarly, coimmunization of the keyhole limpet hemocyanin (KLH) with the BEVs derived from the nonpathogenic *E. coli* W3110 mutant strain induced stronger specific antibody responses and T cell activation than those induced by KLH alone [149]. Apart from these protein antigens, the adjuvant activities of BEVs have also been confirmed for peptide antigens, such as the SIINFEKL peptide antigen [149], while in a further study, coimmunization with the engineered BEVs that express model subunit antigen green fluorescent protein (GFP) maintained a longer immune duration than the *E. coli*-derived BEVs that mixed with the GFP [150], suggesting that the stronger binding of BEVs to antigens might be beneficial to enhance their adjuvant potencies.

Alternatively, the antigens can be exogenously anchored onto the surface of BEV adjuvants by either chemical conjugation or physical insertion. For instance, by covalently linking the capsular polysaccharide of *N. meningitidis* group B (BPS) onto *N. meningococcal* group B M986-derived BEVs, higher levels of the active bactericidal antibodies were elicited than the BPS alone [151]. Besides, by covalently linking the LPS of the *Brucella abortus* S99 onto the *N. meningitidis* group B-derived BEVs, higher levels of the specific IgG antibodies were also found to be induced than the simple mixture of LPS and BEVs [152]. Most notably, a versatile modular platform based on SpyCatcher and SpyTag has been developed to facilitate the customizable and facile ligation of a variety of antigens to the BEVs (Fig. 8B) [153]. Apart from these chemical conjugating approaches, physical insertion approaches have also been employed. For instance, by inserting their lipid part into the membrane of *E. coli*-derived BEVs, glycosylphosphatidylinositol (GPI) was anchored to the surface of BEVs, enabling multiple antigens to be simultaneously modified on the surface of BEVs, providing a modular strategy for the development of new vaccine platforms [154].

In addition, antigens can also be endogenously expressed either at the surface or in the lumen of BEV adjuvants by genetic engineering. The expression of antigens at the surface of BEVs can promote the recognition of these antigens and the activation of specific immune responses. One of the earliest strategies used to express antigens at the surface of BEV adjuvants was based on autotransporters. For instance, using the hemoglobin protease autotransporter platform, 3 *M. tuberculosis* antigens were successfully expressed at the surface of *E. coli*-derived BEVs [71]. Despite its high efficiency, the autotransporter-based strategy only allows the expression of the relatively smaller protein fragments. To express the large protein fragments, the cytotoxin ClyA has been utilized as a fusion partner to express the heterologous proteins at the surface of *E. coli*-derived BEVs, enhancing their potencies as adjuvants [155–157]. Apart from the surface, the antigens can also be expressed in the lumen of BEV adjuvants. As such, the antigens can be protected from proteolytic cleavage to achieve increased structural stability and presentation efficiency. There are two classical strategies to express antigens in the lumen of BEV adjuvants. One is to fuse the antigen proteins with a twin-arginine (Tat) signal sequence so that the antigens can be expressed in the lumen of BEV adjuvants [158], while the other is to fuse the antigen protein with the outer membrane protein OmpA sequence so that the antigen can be expressed at the periplasmic side of BEV adjuvants [159]. To obtain multivalent vaccines, BEV adjuvants with antigens being simultaneously expressed both at the surface and in the lumen have also been constructed. For instance, Wo et al. [160] have used ClyA and OmpA to present two different SARS-CoV-2 antigens, both at the surface and in the lumen of *E. coli* Nissle 1917-derived BEVs, respectively (Fig. 8C). The bivalent antigen display platform could facilitate the acceleration of innovative vaccines.

Besides the protein antigen, the engineered BEVs can also be exploited as adjuvants for the polysaccharide antigens. For instance, Price et al. [161] have constructed the nonpathogenic *E. coli* strain-derived BEVs expressing the surface polysaccharide of the pathogenic bacteria. These glycoengineered BEVs were demonstrated to possess remarkable vaccine potencies against both *Streptococcus pneumoniae* serotype *14* and *Campylobacter jejuni.* In another study, Chen et al. [162] have generated the *E. coli*-derived BEVs expressing recombinant O-antigen polysaccharides (O-PS). The glycosylated BEV vaccine successfully protected mice from the *Francisella tularensis* challenge.

In conclusion, the PAMPs, nanoscale size, and bacterial source of BEVs make themselves promising vaccine adjuvants since they enable antigen display, lymphatic drainage, antigen-presenting cell uptake, and potent immune activation. The vaccine adjuvant effectiveness of BEVs could be further amplified through rational design and engineering. For instance, protein antigens and polysaccharide antigens could be exogenously anchored via physical insertion or chemical conjugation, and could be endogenously expressed on the surface of BEVs or within their lumen. These approaches collectively assist BEVs as effective adjuvants for the development of potent, customizable, and versatile full-functional vaccines to induce strong, specific, and durable immune responses against bacterial infections.

### BEVs as nanocarriers in delivery

**Fig. 9.**
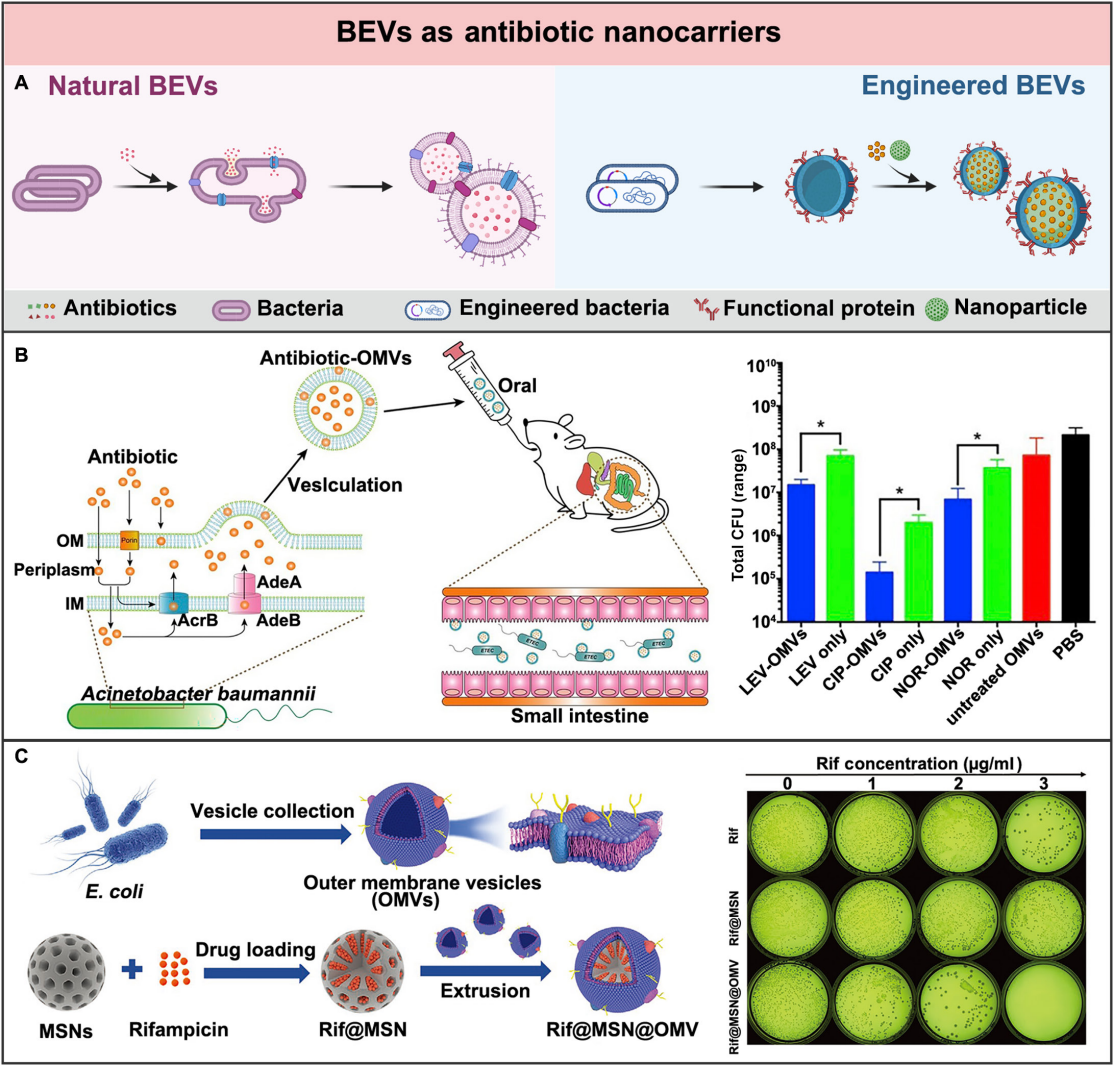
BEVs as nanocarriers in delivery for bacterial infection treatment. (A) Schematic illustration of the comparison of the natural and engineered BEVs as nanocarriers for the antibiotic delivery. Created in BioRender. (B) By using the drug efflux mechanism, *A. baumannii*-derived BEVs loaded with antibiotics were generated, reducing the pathogenic bacterial load in the small intestine and feces of mice. Adapted from Ref. [116] with permission. Copyright 2019, Elsevier BV. (C) Nano-delivery system Rif@MSN@OMV was constructed by coating *E. coli*-derived BEVs onto rifampicin-loaded MSNs, specifically enhancing the uptake of rifampicin only in *E. coli* cells and achieving specific elimination of *E. coli*. Adapted from Ref. [170] with permission. Copyright 2021, Wiley-VCH GmbH.

Antibiotics administered via conventional routes, such as oral medication and intravenous injection, have only a limited utilization rate in vivo [163]. Multiple dosages are often required to maintain their effective concentration and bactericidal effect at the infection site. However, the repeated administration of antibiotics highly increased the risks of their systemic toxicity and bacterial resistance [164]. Therefore, nano-delivery systems have been applied to overcome these limitations. Among all the nanocarriers, liposomes are the most common choice. They can load hydrophilic antibiotics in their lumens while hydrophobic antibiotics are in the middle of their lipid bilayers, protecting the antibiotics from degradation and increasing their concentration at the infection site [165]. With similar lipid bilayer nanostructures to liposomes, the BEVs secreted by bacteria have attracted more and more interest [117,166]. Apart from featured properties like liposomes, BEVs own several unique properties for the exogenous delivery of antibiotics. First of all, BEVs share almost the same membrane components as their parent bacteria, facilitating their fusion with the bacterial membrane and thus increasing their efficiency in the delivery of antibiotics into bacteria. Besides, BEVs inherit quite a lot of bioactive substances from their parent bacteria, which may sensitize or potentize the bactericidal effects of the antibiotics. Moreover, BEVs can also be endowed with additional functionalization based on diverse engineering strategies, fulfilling varied demands during bacterial infection treatments (Fig. 9A).

The earliest research on BEV-assisted antibiotic delivery was reported in 1996. In this research, *P. aeruginosa* was exposed to gentamicin to observe the release of BEVs [88]. The generated BEVs were demonstrated to contain a small portion of gentamicin and exhibited enhanced bactericidal potency compared to either the free form of gentamicin or BEVs without gentamicin. In a follow-up study, these BEVs were furthermore shown to possess strong antibacterial activity against the gentamicin-resistant *P. aeruginosa* [167]. These findings inspired the exploitation of BEVs as nanocarriers for the delivery of antibiotics. Since the efflux of antibiotics by secreting BEVs is one of the mechanisms for bacteria to develop resistance, a passive loading approach was proposed to generate the antibiotic loading BEVs [166]. For instance, Huang et al. [116] have cultured *A. baumannii* in the medium containing antibiotics to passively load antibiotics into the generated BEVs (Fig. 9B). These BEVs facilitate the stable delivery and efficient internalization of antibiotics, thereby markedly reducing the *E. coli* colonized in the small intestine of the infected mice. However, the passive loading approach based on the antibiotic efflux mechanism is not efficient and also not suitable for the antibiotics that can be easily degraded inside the bacteria.

In addition, the antibiotics have also been loaded into BEVs via either genetic engineering approaches or physicochemical engineering approaches. Depending on the various conditions employed, physical engineering approaches can be further divided into sonication, electroporation, and membrane fusion. For instance, BEVs were coated on the lipid nanoparticles that are loaded with the hydrophobic norfloxacin to generate antibiotic loading hybrid vesicles for the treatment of bacterial infections [168]. Further, Li et al. [169] report a multifunctional nanomedicine, in which the BEVs from *E. coli* coassembled with the prodrug composed of two phenylboronic acid-modified ciprofloxacin molecules and one ellagic acid through extrusion.

Apart from antibiotic loading, BEVs have been engineered to achieve specific targeting as well. For instance, Wu et al. [170] have used *E. coli*-derived OMV (BEVs) to wrap mesoporous silica nanoparticles (MSNs) loading rifampicin, generating a biomimetic nano-delivery system, Rif@MSN@OMV (Fig. 9C). These engineered BEVs exhibited high antibiotic loading, narrow size distribution, and long-term stability. Most notably, they also displayed selective bactericidal effects toward the pathogenic *E. coli,* owing to the homotypic targeting capacity of BEVs. Besides, Wei et al. [171] have genetically engineered *E. coli*-derived BEVs to express the anti-Bap protein, which can specifically bind to the surface protein Bap displayed by *A. baumannii*. Thereby, the generated BEVs exhibited selective bactericidal effects against the pathogenic *A. baumannii*, rather than their parental bacteria *E. coli.* Moreover, Peng et al. [168] have engineered BEVs with neutrophil-derived extracellular vesicles (EVs), generating hybridized EVs that could firstly target the inflammatory microenvironment. Thereby, the accumulated EVs at the infected site could further target specific bacteria, suggesting their great potential for the treatment of systemic infections.

In conclusion, the lipid bilayer, nanoscale structure, and bacterial membrane composition of BEVs facilitate their capacities as antibiotic nanocarriers. These properties could be further optimized through rational design and engineering strategies. For instance, all genetic, physical, and chemical engineering approaches could assist BEVs in loading antibiotics with high efficiency and stability. Besides, genetic engineering of BEVs could also enable the expression of targeting ligands and the precise delivery of antibiotics to the specific infected sites or pathogens. Moreover, material engineering, such as coating BEVs onto nanoparticles and fusing BEVs with other membrane vesicles, could generate hybrid systems as well with enhanced antibiotic loading efficiency, controlled size distribution, and customizable functions. Compared to the natural BEVs, these engineered BEVs with controlled particle size, high antibiotics loading, and customizable targeting capacity offer more possibilities for the effective treatment of diverse bacterial infections.

## Perspectives and Outlooks

**Fig. 10.**
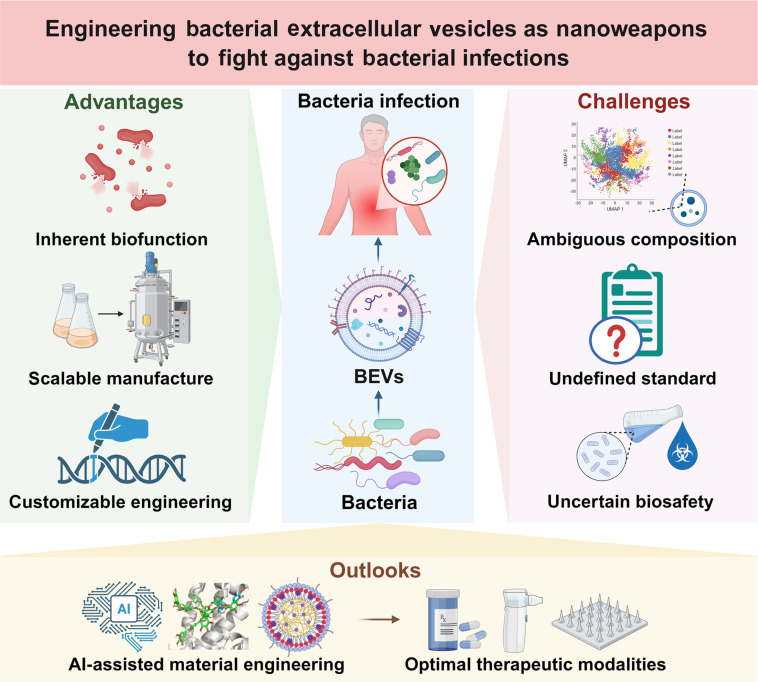
The advantages, challenges, and perspectives of engineering the BEVs as nanoweapons to fight against bacterial infections. The BEVs possess several advantages, including the inherent biofunction, scalable manufacture, and customizable engineering for the treatment of bacterial infections. They also face several challenges, including the ambiguous composition, undefined standard, and uncertain biosafety, to completely unleash their therapeutic potentials. In the future, the AI-assisted material engineering that rationally combines multiple functionalization strategies is expected to be exploited for tailoring BEVs as optimal therapeutic modalities to achieve satisfactory therapeutic outcomes of bacterial infections. Created in BioRender.

BEVs are nanosized vesicles with lipid bilayer membranes that are secreted by bacteria. Owing to their structural and componential similarities to the parental bacteria, tremendous efforts have been devoted to exploiting BEVs as nanoweapons to fight against bacterial infections (Fig. 10). For instance, since BEVs transfer various antibacterial molecules, such as antibacterial hydrolases, peptides, and metabolites, from their parental bacteria, they have been directly used as antibacterial agents to kill pathogenic bacteria. Besides, due to the surface proteins, including the adhesins that the BEVs inherit from their parental bacteria, they have been directly used as anti-infection agents as well to competitively inhibit the adhesion and infection of the pathogenic bacteria to the host. Moreover, with the emerging engineering approaches, additional capacities have also been endowed to BEVs, further broadening their roles for bacterial infection treatment. For instance, based on either genetic or chemical engineering approaches, the surface components of BEVs, including their inherited PAMPs and pathogen-specific antigens, can be rationally modified. As such, these BEVs have been engineered as either vaccine antigens or adjuvants with the appropriate immunostimulatory effectiveness for the effective and safe treatment of bacterial infections. In addition, based on genetic or physical engineering approaches, the internal contents of BEVs can also be effectively adjusted. Thereby, these BEVs have been engineered as nanocarriers as well with the desired targeting and penetrating capacities for the optimal treatment of bacterial infections [172,173].

Compared to conventional antibacterial and antiadhesion agents, bacterial vaccines and adjuvants, and nano-delivery carriers for bacterial infection treatment, BEVs possess several superiorities. First of all, BEVs themselves are bioactive owing to the internal substances, surface components, and lipid bilayer nanostructures they inherited from their parental bacteria. Secondly, the manufacturers of BEVs are scalable due to the rapid proliferation and mature culture procedures of bacteria. Thirdly, the modifications of BEVs are customizable thanks to the advanced genetic, physical, and chemical engineering approaches. These superiorities of BEVs offer more possibilities to meet the various demands during the different bacterial infection treatments. With the successful commercialization and clinical use of Bexsero, a BEV-based vaccine to prevent meningococcal group B diseases caused by *N. meningitidis*, more and more BEVs that have been developed to fight bacterial infections are expected to be pushed from bench to bedside in the future [174].

Despite these superiorities of BEVs, there are still several challenges that need to be tackled to completely unleash their therapeutic potential for bacterial infection treatment. First of all, even though the bioactive cargoes of BEVs have been gradually identified, most of them remain ambiguous, leading to unknown and uncontrollable clinical application risk. Benefitting from the high-throughput omics technologies, several databases, including Vesiclepedia, ExoCarta, and EVAtlas, have already been created as compendia of these biomolecules in exosomes. Nonetheless, a similar database specialized for BEVs is still lacking. Based on the rapidly advanced artificial intelligence (AI)-driven multiomics analyses and machine leaning algorithms, the genomics, proteomics, and metabolomics of BEVs could also been systematically integrated, assisting the establishment of the BEVs database. Secondly, enormous variations exist in the structure and composition of BEVs derived from the different bacterial strains or even the same bacterial strain with different culture conditions or isolation techniques. These heterogeneities of BEVs seriously complicate their consistent therapeutic performances. However, the international unified manufacturing workflows and evaluation standards are still in high demand to guarantee the precise therapeutic outcomes of BEVs. It is crucial to standardize upstream processes, such as utilizing the fully sequenced and characterized bacterial strains as well as employing the defined and optimized cultivation parameters. Meanwhile, it is also necessary to replace or supply the UC-based isolation with more consistent and scalable techniques, and define strict BEV purification protocols. Thirdly, rather than completely knocking out genes and removing immunogens, the attenuated bacterial strains have usually been engineered to generate BEVs with optimized immunogenicity. As such, the appropriate immunostimulatory capacities of these BEVs have been preserved with reduced biotoxicity. Even so, the long-term biosafety profiles of BEVs still need further investigation. Meanwhile, the evaluation criteria regarding the immunogenicity and biotoxicity of BEVs should also be established to ensure their effective and safe treatment of bacterial infections. A multitiered and quantitative assessment framework involving the detailed immunoassay alongside the system toxicity monitoring both in vitro and in vivo are needed to balance the immunostimulatory capacities and biotoxicity of BEVs. Repeated-dose toxicity studies in relevant animal models or long-term coculture with human immune organoids could also provide insights into the long-term safety assessment of BEVs. Furthermore, the immunostimulatory spectrum and potential biotoxicity of BEVs could be predicted as well with the rapidly advanced machine learning based on the BEVs database in the future.

With the emerging cutting-edge technologies, the administration routes of BEVs should be tailored as well according to the different sites of bacterial infections to achieve the optimal therapeutic outcomes [175]. For instance, the BEVs engineered in inhalable formulations are more suitable for the treatment of lung infections, while those engineered in microneedle devices are more desirable for the treatment of wound infections [176,177]. Thereby, apart from the conventional genetic, physical, and chemical engineering approaches, the material engineering approaches that combine multiple functionalization strategies should also be proposed and applied to construct the optimized therapeutic modalities. Furthermore, machine learning and AI should be exploited as well to guide and boost the rational design and engineering scheme of the BEV-based therapeutic modalities. For instance, Chouaib et al. [178] have developed the Intelligent Vesicle Exocytosis Analysis platform, which might be extended to predict the most effective BEV subtype for facilitating their intracellular uptake, assisting the design guidance of the BEV-based precisive medicine for bacterial infection treatment. Integration of the AI technique with BEV engineering can also provide the possibility to circumvent the translational barriers regarding the BEV-based bacterial infection treatment including loading efficiency, targeted delivery, in vivo stability and pharmacokinetics, and immunogenicity and safety [179]. Since the performances of the deep learning- and machine learning-based AI techniques are contingent on the data quality, regulatory frameworks and standardized procedures should be proposed in the future to guarantee the credibility and reliability of the data collected. These integrated interdisciplinary sciences and technologies are envisioned to work out the most powerful nanoweapons based on BEVs for conquering bacterial infections.
